# Listening to face transplant patients and caregivers: how medical humanities approaches redefine surgical ‘success’

**DOI:** 10.1136/medhum-2024-013005

**Published:** 2024-12-19

**Authors:** Fay Bound Alberti, Dallas Weins, Annalyn Bell Weins

**Affiliations:** 1 History, King’s College London, London, UK; 2 Fort Worth, Texas, Forth Worth, Texas, USA

**Keywords:** Ethics, cultural history, patient narratives, Medical humanities, psychology

## Abstract

A recent review of face transplants argues that overall, they have been successful. But this verdict is based on surgical measures rather than patient-reported outcomes (PROMs), which for historical reasons are in their infancy. These measures are critical to understanding the nature of success in face transplants, and the evidence from mixed systems of healthcare, as in the USA, reveals that there are significant ethical and social concerns about the well-being of patients. Medical humanities research that focuses on the lived experience of patients and their caregivers can contribute significantly to the discussion by focusing on patient voices and the measures that matter outside of surgical contexts. This article builds on existing work and original interviews with face transplant recipients and their families from an emotion history perspective. It argues that surgical measures used in isolation can be misleading. We need a more holistic understanding of outcomes—financial, psychological and emotional as well as medical—that requires the insights drawn from the humanities and transforms the definition and measurement of ‘success’.

## Introduction

 The first face transplant (FT) was carried out in France in 2005 and the procedure was subsequently developed across several countries with different healthcare systems (Bound Alberti 2017; Bound Alberti and Hoyle 2021). FT are highly complex and expensive procedures that involve considerable medical and psychological risks and require life-long follow-up and care. The most recent assessment of the success of FT, in *JAMA*, concludes that ‘the acceptable long-term survival of face transplants makes them an effective reconstructive option for extensive facial defects’ (Homsy *et al* 2024). One could debate that claim in surgical terms —of 50 transplants, two people needed to be retransplanted, patients experienced an average of 1.2 acute rejection episodes a year; and 10 patients died, which is a relatively high proportion (20%). However, this article focuses on the broader question of emotional, social and financial health and PROMs, which are the key interests of the Interface project.

Interface is a major interdisciplinary research project, funded by UK Research and Innovation (UKRI) through a Future Leaders Fellowship (FLF). The project evaluates the history and meanings of FT as a visible form of VCA (vascularised composite allograft, meaning the transfer of multiple tissues— skin, muscle, bone, nerves, blood vessels and connective tissue, from a dead donor to a recipient as a functional unit). Interface uses historical methodologies (especially from the histories of medicine and emotion) and insights from sociology, anthropology, literary theory and gender studies, to offer a more expansive and holistic approach to FT than has been developed to date. It does so within the broader context of cultural attitudes towards the face as an emotional and symbolic part of the human body, associated with legal and personal identity and self-hood as well as being a sensory, communicative space.

Interface works with patients and surgeons as well as humanities scholars and social scientists. In 2021 it convened (as AboutFace, based at the University of York) an international, interdisciplinary Policy Lab at King’s College London to examine how best to tackle these issues and include them in a sustainable FT framework. The Policy Lab findings were made available for free online, in keeping with the principles of UKRI funding,and an Open Access article was published with surgical contributors (Longo *et al* 2024). The Policy Lab brought surgical teams from around the world together to recognise the key challenges, share data and reach a consensus on what needed to be improved in FT—as well as what success might look like. This is important because although relatively few FT have taken place, *how* they take place has transferable implications for all forms of innovative surgery. For individual patients and their families, FT are transformative in positive and negative ways.

As Policy Lab participants agreed, data on long-term clinical and quality of life (QoL) outcomes are lacking and outcomes not routinely shared between programmes. To date, no universal standards of care or agreed metrics of success for FT exist (Bound Alberti *et al* 2022; Fullerton *et al* 2022). Without that data it is difficult to determine how success is measured, whether FT deliver more satisfactory results than traditional facial reconstruction or how they might be compared with future interventions like tissue regeneration. More generally, it is a problem that FT outcomes are not compared with other possible avenues for patient care including prosthetic use. The history of medicine shows that it is the innovative and the radical that tend to get media attention and financial investment. This is part and parcel of how progress in scientific medicine has been defined as the largely individual work of path-breaking innovators. Behind every innovation, of course, there are ethical uncertainties; results are not always as planned and those who are most affected can be left behind.

In the field of FT, more work needs to be done to determine what matters to patients as well as their caregivers. FT produce a highly visible ‘before and after’ with cultural resonance as a form of transformation; there are far more symbolic meanings than in the transplant of ‘invisible’ organs, such as a kidney or a liver. (Bound Alberti 2020). The only comparable organ is the heart (Bound Alberti 2010). And besides the cultural meanings of the face, there are ethical implications to FT—including the creation of a ‘permanent patient’, thanks to the side effects of the immunosuppressants and the ongoing level of care needed. The need for lifelong immunosuppressants is generally accepted in solid organ transplants which are lifesaving. However, FT are not life-saving; they are life-enhancing and intended to restore a person’s social relationships as well as their facial functions. This social context is, in addition to the medical risks, why it is so important for patients to have a strong support network—and most institutions assess this as part of their evaluation process. Yet though a person’s social network might be evaluated before the transplant, as well as their access to financial and other material resources, these factors tend not to be evaluated on an long term basis. Given the likelihood that patients will have ongoing health problems and challenges that place stress on their emotional and social resilience, postoperative support needs to be built into FT from the planning stage to delivery and the longer term.

There has been some recognition from surgical teams that the current way of determining outcomes is not sufficient. But it is difficult to make headway, especially from other disciplines; critical evaluations of FT from a medical humanities perspective are often rejected by surgical journals like *JAMA*—either because the data is not considered ‘scientific’ because it is drawn from qualitative rather than quantitative sources or because surgeons who are nominated as peer reviewers take the perceived criticism poorly and sometimes personally. That is not the intent of Interface which is principally concerned with the limitations of healthcare systems rather than individuals and with showing how qualitative data can influence the questions being asked, as well as the framing of outcomes. Since Interface is committed to making space for patient voices, and no patients were able to be present at the Policy Lab, this article has been written as a collaborative enterprise between myself, as head of the Interface project, Dallas Weins, the US’s first full FT recipient, and Annalyn Bell Weins, Dallas’ wife and caregiver.

Of the several themes addressed by the Policy Lab (that included surgical evaluations and lessons learned for operating teams), the following were seen as most pressing in the global landscape of FT: patient selection and evaluation, family, social and peer support networks, and financial sustainability, all of which will be considered in this article. Some of these concerns—notably financial sustainability—are more relevant to some contexts than others. In countries like Finland where patients receive long-term holistic care due to a state-funded healthcare system, patients have more positive responses to the question of long-term care than in mixed healthcare systems. The patients interviewed for this paper (five men and one woman) are all based in the US where commercial interests dominate.

## Materials and methods

The Interface project has the ethical approval of the UK’s Health Research Authority. This is a qualitative research project that collaborates with nine international surgical teams and six National Health Service sites. This article has been informed by semi-structured narrative interviews with six FT recipients, two of whom wish to be named, as well as five members of their friends and family networks; two ethicists, three psychologists and six surgeons involved in FT; and the data generated by the Policy Lab. That data has been analysed using a mixed methods approach with insights derived from the histories of medicine and emotion. For ethical reasons, no family members under 18 have been interviewed, and no patients under 18 have ever received FT. The following discussion has relevance to all forms of VCA but especially hands and faces as visible forms of transplantation that are intended to enhance, rather than save lives.

### Patient and public involvement

Patient and public involvement is core to the Interface project and this paper is based on detailed interviews with patients and their caregivers. None of those patients are referred to or identifiable by name in this manuscript with the exceptions of Dallas Weins (coauthor, now deceased) and Robert Chelsea who has taken a public role in talking about his FT to promote organ donation in black communities. For more information see: https://www.robertchelsea.org

## Discussion

### Patient selection and evaluation

When a patient undergoes FT from a compatible donor, that is not only because they have a form of facial difference (or ‘disfigurement’ to use the legal and medical term) for which the procedure has been deemed medically appropriate. Patients need to have demonstrated psychological ‘resilience’ and to have shown no contraindicating health problems. And they must – to comply with informed consent – possess a thorough understanding of the risks and the benefits, as well as a willingness to be compliant with the necessary medication and lifestyle, and have a good support network. How far these factors are evaluated varies, and there is also variability in who is seen as an appropriate patient. Not all clinics accept those with a history of attempted suicide, for instance.

The Interface project has shared data with clinics on how humanities perspectives might influence patient evaluation through QoL questionnaires (including with the Brigham and Women’s Hospital in Boston, where the two patients cited in this paper, Dallas Weins and Robert Chelsea, both received FT). It has also explored with clinics how psychological and social resilience could be tested. From a history of emotion perspective that means considering how people feel about their faces, rather than presuming they will have poor emotional health or low self-esteem. Attitudes towards appearance are complex; people with acne can have poorer self-image than those with severe facial injury. Patient consultations need to involve a person’s wider family and support networks and share all available data with patients including that which might be off-putting. Without it, it is impossible for patients to make fully informed choices.

Historians of medicine and emotion know that there is nothing transparent or objective about patient evaluation processes. Healthcare often involves power dynamics and hierarchies as well as intense emotional experiences, meaning that patients and families are enmeshed in complex relationships—between patients and surgeons, within extended surgical teams, and between hospital administrators and funders, all of whom have their own motivations and incentives (Sebring 2021). This is particularly notable in the US context where leading specialists are described in promotional and conference literature as ‘rock star surgeons’. Patients, especially when vulnerable, worry about expressing their concerns and being labelled ‘bad patients’—or worse, ‘ungrateful’ which is a commonplace fear when it comes to transplants because of the emotional framing of organ donation as a gift. The language that we use matters to the power that people think they have in medical settings and to the ways in which agency is negotiated (Angeli and Johnson-Sheehan 2018). Surgeons also have their own emotional influences, no matter how far the field has historically presented itself as objective (Brown 2022; Sharp *et al* 2020). It is important to acknowledge these often conflicting relationships, and the emotional entanglements that are involved (whether that’s an individual surgeon’s desire for promotion or a clinic’s need to secure grant funding or a patient’s emotional dependency on a caregiver). Medical decisions involve human actors and are not objective.

All patients acknowledged that they were informed about the risks of immunosuppressants, death and negative outcome consequences. Yet all also recognised a gap between theory and reality; hearing that one might undergo kidney failure or graft rejection alongside a list of possible outcomes at the evaluation stage is not the same as experiencing those problems years into the transplant when a person has already undergone multiple episodes of rejection, isolation and financial hardship. That emotional stress is felt not only by patients who monitor themselves for rejection but also by their families and caregivers who worry about their loved ones. An evolving notion of consent is needed; one that continually feeds back to the surgeons on patients’ well-being, is adaptable in making necessary changes, and can make that data available for future patients. At present, prospective patients might be introduced to FT patients who are enjoying positive outcomes but not to those who decided against having a transplant or are experiencing rejection and other negative outcomes.

In 2011, Dallas Weins underwent the first full FT in the US, three years after he was electrocuted in an injury that destroyed his eyes and his face. He found the assessment process “incredibly informative. [My surgeons] were upfront and straight and honest about the damage the medications could do to my kidneys. They were very clear that because I was so young, you know, I had both my age working for me and against me because I would be on immunosuppressants for so long”. Immunosuppressants prevent the body’s immune system from attacking the foreign facial tissue, but they have significant negative effects on the rest of the body by exposing the patient to cancers and kidney failure. Patients must take immunosuppressants daily and for life.

Dallas’ decision to have surgery was influenced by his positive experience of meeting with a FT recipient with good outcomes, and he has been invited to meet with other prospective patients in turn. This personal sharing of experience formed an important part of the evaluation process for him as a patient, irrespective of the role it had for the surgeons and he believes it influenced at least one other patient. “I was just honest… about how I felt, you know…And…because of that communication, he got to get his face transplant done”. Yet, with full and informed consent as part of the evaluation process, patients should have access to a wider range of personal experiences including those that were negative. More than a decade after his transplant, Dallas reflected that:

“If I could have spoken to somebody who was going through what I am going through now [kidney failure], it would have impacted on my decision to even get the transplant. It doesn’t mean I wouldn’t have gone for it. But I would have clearly taken a step back and given it more consideration over the long term. Before opting in to being put on the waiting list.”

When Dallas started to experience significant health difficulties, he found that he was not brought in to meet other patients, and believes this was a missed opportunity: “now if I were to talk to a patient, I’d still be completely honest about what I’m dealing with, and be a little more reserved with the idea should I do this or should I not?” All patients interviewed for the Interface project agreed with this perspective, but it tends to be most expressed around five years after FT when patients tend to experience more frequent negative symptoms. It is important to note that the average life expectancy of a FT – based on solid organ transplants – is usually given as ten years. Since 2005, two patients have been successfully retransplanted, but most patients do not have a plan b. Dallas’ surgeon took precautions to ensure some of his own face was left should the worst happen; not all surgeons are equally conservative, especially when their goal is to push the envelope of experimentation.

The Policy Lab delegates observed that prospective patients tend not to be presented with a wide range of alternative treatment approaches. There is no comparative data available for FT versus prosthetics outcomes in terms of quality of life, for instance, or FT versus traditional reconstruction that doesn’t require immunosuppressants. For truly informed consent, a patient should be given a full list of pros and cons for each procedure, as well as introductions to patients who have undergone them. It skews the evidence if prospective patients are only shown the good and not the bad, and it denies learning for the FT team as well as the patient and their family.

This concern that patients are provided with the full picture connects to the importance of data sharing between international teams. There is no reason why (with properly managed consent and privacy) anonymised outcomes should not be shared internationally—at least from an ethical perspective. It is the case, however, that surgeons tend to downplay negative outcomes publicly, since grant funding and reputations are at stake. Given the relatively small number of clinics around the world who are specialists in FT, accurate and full data sharing would support the development of international standards. It would also encourage surgeons to look beyond the immediate priorities of individual teams. At present, there is no mandatory and regulated process by which positive and negative outcomes are recorded, and what is recorded – as we will see – can be misleading.

All patients interviewed felt that aftercare was the biggest drawback from their clinical experience. In the US context, there are ethical and practical failings in the psychological support provided for patients, in the financial support given for patients to travel to appointments, and in the communication between caregivers and the hospital, and between the hospital and primary care physician (PCP—equivalent to a General Practitioner or GP in the UK). In part, this lack of joined-up care reflects the highly specialised nature of the procedure and the different standards of support available locally. Yet the disappointing truth is that there is more sustained effort put into evaluating patients for FT than in ensuring long-term care, and more grant funding for experimental procedures than for existing patients.

“My surgical team in Boston were very clear with me on what I can and cannot expect over a specific period between three and five years”, Dallas reported. “They were clear that [nerve function] would not be 100% because there were nerve clusters that were too damaged to exist. And they explained about the risks, including kidney failure… So, what they failed me on was all the aftercare.” [In the beginning the hospital would] “fly me up there on a regular basis and do all the exams and pay for everything and all of that, but it started to fade over time. I would go up less and less often. First, they couldn’t repay everything, then they weren’t able to do anything at all.” The need for a joined-up response to aftercare is particularly important because FT expertise is clustered into a few centres and travel is inevitable for patients. Dallas’ concern that aftercare ‘just kind of slid off’ is common from a wide range of conversations with patients and their caregivers. Before any experimental surgery like FT can become a standard of care (and before it can benefit from medical insurance funding), it needs to demonstrate improved QoL and better outcomes for patients than other treatment options. And that has not yet happened.

### Family, social and peer support networks

The need for a strong social support network came out clearly from the Policy Lab and from Interface’s research with patients and caregivers. It is also widely recognised by FT clinicians that family and social support is integral to patient selection; without that support patients will not have their long-term emotional, social and financial needs met. However, it is challenging to understand and evaluate support networks for a variety of reasons.

Firstly, people who have undergone significant facial trauma might develop coping mechanisms – including drug and alcohol dependency – that make it difficult to sustain healthy social and family relationships; sometimes the trauma and dependency are consequences of those relationships (Kumnig and Jowsey-Gregoire 2015). Some FT recipients interviewed by Interface reported addiction problems with drugs and alcohol as survival strategies that ultimately alienated them further from their families and caregivers. This is further evidence that a longer-term approach is needed to assess support networks. That which might be in place in the immediate or short-term aftermath of a life-threatening injury may fall by the wayside under financial and emotional pressures. We are not all blessed with caring and loving families, whether we require a FT or not, and recognising that is critical to an equitable approach to healthcare. Strong support networks should not be regarded as separate to medical care, but as an underpinning branch of it. After all, people are far more likely to indulge in behaviours that, clinically speaking, make them ‘non-compliant’, from drug use to numb emotional pain, to forgetting or avoiding taking medications, without practically- and emotionally-supportive human relationships.

Secondly, assessments of loving and supportive family and social networks are not value-free exercises. Robert Chelsea, the first African American individual to receive a FT (and the oldest person to date) has spoken about the hidden racial dynamics in his own assessment—there was an implicit presumption, he has said, that as a Black man he was less likely to benefit from the kind of supportive family structures taken for granted in white families. That characteristic of healthcare assessment is not limited to FT but also found in solid organ donation (Jindra *et al* 2021). Additionally, Robert found the measurement of his emotional state after his injury (including the likelihood that he was suicidally depressed) skewed by racist medical structures. Black people have far lower incidences of suicide and suicidal ideation than white people. And yet Robert was repeatedly asked whether he was suicidal because of the way that he looked. Gendered presumptions about women’s family roles and support systems similarly come into play as women are widely expected to have better emotional resources than men *and* a greater need for a pleasing aesthetic appearance. These hidden presumptions about race and gender need to be made explicit as forms of unconscious bias during the evaluation and selection process.

Peer support is also important. For FT recipients as well as others who have undergone VCA– like hand or penis transplant recipients – learning from and sharing with those who have gone through similar experiences is crucial for emotional insights and a sense of collective identity. This has been widely recognised as important to the wellbeing of solid organ transplant recipients and their families (Joo *et al*. 2022), but there is an important caveat - any peer support network should be run by and for the patients and caregivers. It is ethically inappropriate for them to be managed and data mined by other parties, whether they are hospitals and institutions or individual researchers and groups with a vested interest in using the information shared. Interface has spoken to several patients who would like clinics to take the lead on establishing peer support networks and some patients have individually reached out to other patients, thus avoiding some of the privacy and safeguarding concerns. Dallas is one such person: “some people need encouragement to move on from being a victim to being a survivor”, he says, noting how often formal psychological support is missing. “And I would be quite happy to give them that. To encourage them. But it doesn’t look like the hospital is going to facilitate something like that”.

It is not inconceivable that hospitals could facilitate this kind of resource, though appropriate regulatory frameworks would need to be in place to ensure that patients are safeguarded and not inappropriately responsible for the complex emotional needs of others. From the perspective of caregivers, too, this kind of support is critically needed—again as evidenced by what is available in cases of solid organ donation (McKinney *et al* 2021). Annalyn Bell Weins is Dallas’ wife and principal caregiver, and like Dallas visually impaired. She believes that it would be invaluable for carers to learn from others who have gone through similar experiences:

“Like, what do you do? What have you found to work? What are we looking at? What’s the experience going to be like? That kind of thing. You can share your own experience. I’m moving into this blind”, Annalyn says, “literally, and also, figuratively. So, it would help…[and] it would be just as productive as having a communication line for patients to talk. That would be a phenomenal help to caregivers. Truly.”

The emotional and practical energy involved in caregiving—not only in monitoring the health of patients who end up with complex medication and healthcare regimes, but also in guarding against errors in medication that are prescribed by local pharmacies, mediating between patients and hospitals, knowing what is available for patients and supporting them financially and practically—takes an enormous toll. We need sustainable and joined-up systems of care to take the pressure off individuals caregivers. It is likely that fear of litigation (and perhaps a desire to prevent patients from sharing negative outcomes in ways that are detrimental to hospitals and surgeons) prevents hospital sponsorship of peer support networks. Yet providing more extensive support for caregivers and patients would alleviate many day-to-day struggles and reduce isolation.

Well-established peer support networks would also empower caregivers to resist the power imbalances that can be at the heart of medical encounters, with medical professionals using language and equipment that is removed from the everyday experience of most people. We must factor in, too, the inevitable sense of powerlessness that comes with being a patient not receiving the healthcare they wish for, which sits at odds with the sense of obligation and gratitude that accompanies life-saving or life-enhancing treatment. As with patient evaluation, there are hierarchies at work in medical contexts that impact on how people with disabilities are perceived. Because Annalyn is, like Dallas, registered blind, she feels that she is often patronised and looked down on by clinical teams, rather than being treated as a responsible carer. This is exacerbated by presumptions about gender and age:

“I should not have to dress up extremely professionally… like I’m rich to get the attention of doctors to take me seriously. Because if I’m just in a T-shirt and I’m sitting there with him, and obviously it’s in their files that I’m his medical power of attorney/next of kin, yes you should be taking me seriously, but then I just look like a little girl. Little blind girl… When they come in and they see you’re blind, like, this nurse was introducing herself because I need to go downstairs to get some fruit, because that’s all Dallas could eat at the time. And that’s all he wanted at the time. And she was walking with me, and she was starting to talk to me like I was a child. Like I was a little toddler who couldn’t look after my husband.”

It is common for people with disabilities to feel their capability is questioned by able-bodied people and there are additional complex issues for caregivers with disabilities in medical contexts. Hospitals and surgical teams need to be able to accommodate people with a wide range of abilities. The aftercare, as Annalyn puts it, “needs to focus on all aspects of everybody, of every single caregiver, basically, no matter their circumstance”. And this is important because families and caregivers are likely to change, along with the level and kind of support available. So, a holistic approach to aftercare must recognise the individual needs of patients and their networks, and be flexible enough to measure and evaluate them over the long term.

### Psychological well-being

Policy Lab attendees recognised the importance of psychological health principally in relation to patient evaluation and determining whether individuals were ‘resilient’ enough for the procedure. This has been an ongoing theme of ethical and psychological discussions around FT. In the early days, clinical teams wanted to demonstrate to ethicists and psychologists that FT recipients were aware of the risks and of the emotional consequences of a literal change of face (with all the corresponding identity conflicts that were anticipated). Accordingly, considerable effort is spent by teams specialising in FT to ensure that psychological protocols are adhered to—though that is more thoroughly and authentically carried out in some cases than others.

Regrettably, there is often a short-term view of psychological well-being even though the need for psychological support does not end in the operating room. Most patients who have undergone FT lament that there is very little or no psychological support available over the longer term; as noted, this is particularly likely in US contexts by contrast to countries like Finland where there is a state-funded healthcare system. It is also inevitable that as time passes the emotional needs of patients will change and potentially worsen, especially as significant side effects including chronic rejection come to the fore.

It is imperative, therefore, that psychological well-being is addressed as an ongoing requirement that impacts on the success of facial transplantation. Poor mental health not only damages the well-being of the patient and their caregivers but also makes it more likely that patients will fail to take medication and engage in behaviours that are not conducive to the survival of the graft—which is the most important issue surgically.

Dallas is one of many patients who has been unable to access psychological support for himself or for his family. He worries about the impact of loneliness and isolation that can come from the negative emotional and physical effects of transplantation, too, for himself and for others:

“There was a transplant patient, VCA transplant patient like me, near us somewhere in Texas. And he ended up shooting himself because he was completely alone. I don’t know if it was a full or a partial, or whatever, it was a VCA transplant. But he was completely alone, he had no one to talk to. He couldn’t understand why he was not feeling better. You know, he was feeling ill all the time. Like he just didn’t get it. He couldn’t get over it. And he ended up taking his own life. And it would have been absolutely no problem for [the hospital] to tell him, hey, here’s a phone number. Call this guy.”

There are reasonable ethical reasons why hospitals would not reach out to patients to provide emotional support to others, but Dallas’ concern highlights the fact that psychological problems are commonly talked about between FT recipients. Dallas talks openly about his poor mental health and the barriers in securing therapy. “You have a psychological evaluation before you can be placed on the waiting list”, he says but “that’s literally the last time you talk to a psychologist”. When Dallas tried to find his own psychologist, he found the costs were prohibitive. He was prescribed Zoloft for his depression by the nurse practitioner (NP) of his PCP which he was taking for about a month before he was able to find a psychiatrist nearby. That psychiatrist apparently told Dallas that Zoloft interfered with his antirejection medication and gave him a different prescription. But when Dallas checked with his NP, he was told it was the new prescription that would interfere with the antirejection meds, not Zoloft.

Dallas is sceptical about the motivation of the psychiatrist prescriber in the context of financially incentivised medications with ‘kickbacks’ derived from prescribing a new medication. Whether or not it was a genuine mistake, Dallas believes that “it’s not hard to type a few keys and click a few buttons on a computer to check for drug interactions”. Certainly, the task is not one that should be left to patients and caregivers. The upshot of this lack of holistic care is that Dallas still does not have psychological support, and what he really needs is “a psychologist well versed in trauma, both childhood trauma and general trauma”.

The stories of Dallas and Annalyn, as those of many other patients and their caregivers, chart an exhausting tale of lobbying and petitioning for good quality care within systems that – despite the efforts of a core number of dedicated medical professionals – are not set up for multidisciplinary or holistic practice.

This is important enough to reiterate—Dallas’ frustration is with the system of care that prevails in the US, and that impacts on experimental procedures like FT, rather than with his medical team. Other patients are more critical of their surgeons, especially when an overt desire to innovate seems to dominate the level of care received; once another, more radical and perhaps career-enhancing patient appears, existing FT recipients can feel passed over. Again, that is influenced by funding models and systems, and cultural expectations of medical innovation, but it is ultimately determined by the attitudes and behaviours of individual surgeons.

### Financial sustainability

When I ask Dallas and Annalyn what the most significant problem is that they have encountered, they say the same thing every FT patient interviewed says: “financial, obviously.” For Dallas and Annalyn, the situation is particularly challenging “because we’re both blind. Transportation is a big issue”, Dallas says. “And not one that’s being looked into. The only kind of transportation the hospital suggests is public transportation. And I did that. And the time I did it, I got so damned sick, I refused to do it again”. This is not unusual; all the patients interviewed for this paper reported difficulties in getting to their appointments. Hospitals tend to expect patients to take public transport and that exposes them to the viral load of others which can be fatal especially in the post-COVID world. Taking public transport is part of the resumption of ‘normalcy’ expected from FT patients.

But ‘normal’ is not possible for people like Dallas. Of his post-transplant health when compared with his pre-transplant health, he says that “100% now is the same as 60% then, when it comes to how I feel. Obviously, I’m going to get ill far more often than other people are because my immune system is shot…But then now they’re saying ‘well, won’t you try again?’ Well, no I’m not going to try again. My kidneys are shot. My immune system is still suppressed. Like—can you not help with a $12 Uber ride?”

The financial and logistical challenges of transport are complicated by the distance Dallas and Annalyn live from the hospital—even to make a 45 min taxi ride to a local hospital that can coordinate with the Yale Hospital in New Haven. “That’s $50 or more one way, not two ways. I don’t have $100 dollars for an appointment”, Dallas says. If he does not attend, it can be read as non-compliance but is that fair? In reality, “it’s the fact that we don’t have the amenities to do what they need us to do”.

Dallas had his historic transplant in 2011, and it was the first full FT in the USA. One might imagine that means he provides invaluable data and experience of what it is to live with a transplant long-term, and that he would receive additional monitoring. But he is still struggling to pay for the essential drugs that will prevent his transplant from rejecting. DW’s primary medication alone costs US$120 per month per script:

“I’m on disability now and nobody wants me. And even if I did get a job, I could only make a certain amount, or I would lose my disability. You know we have to tighten our belts and pull up our bootstraps to make sure that we make it through the month every single month. And any stress there could be alleviated if there was some kind of agreement that could help me with, like, I don’t know electricity alone, or like utilities, or part rent. And honestly, with the face transplant community being so small, if only the hospital could get a grant to help us with our financials. That would last multiple years before they have a whole lot of people that they’re having to help.”

Grant funding tends to be won for new and exciting projects rather than supporting existing patients, and that needs to change. In the US most FT have been funded by the Department of Defense as experimental treatments and the long-term health of patients are not included in the predicted costs. And yet no rock star surgeon would be able to push the boundaries of surgery without the consent of patients, and their willingness to undergo life-risking procedures. This point was made by Robert Chelsea at a recent National Academy of Sciences, Engineering and Medicine (NASEM) committee on the subject (National Academies). “We need to be at the table”, Robert says, “just like [the surgeons] are at the table. Because who can give better insights than patients?” He describes patients as ‘case studies’ or ‘specimens that are being observed’ but who are receiving no support for their contribution—unlike everyone else involved in FT who are receiving salaries or grant funding. And “who will pay for our children to be raised [while we are] being observed?”

## Conclusions

As the evidence presented in this paper makes clear, we need more nuance in how FT success is measured and evaluated, from the selection of candidates to their care and aftercare. There is nothing presented in this paper that should be beyond the realms of reasonable ethical expectation or the scope of current surgical treatment. The experiences of patients and their caregivers need to be properly understood and accommodated into medical systems that for too long have not considered those voices as critical to the definition of success. A longer-term view of the well-being of patients that is attuned to the importance of emotions and language, and to the outcomes reported by healthcare users, is essential to determining whether FT can be regarded as ‘effective’ as a form of treatment. Medical humanities research can enrich surgical understandings, offer new insights and ensure that patients get that seat at the table.

### Postscript

On 27 September 2024, Dallas died at his home in Fort Worth. There was no autopsy, and his death certificate states the cause of death as the original accident that gave rise to his transplant in 2011: that is, ‘Complications due to electrocution’.This misleading statement draws attention to the challenges addressed by this paper, and the importance of accurate record keeping and data sharing. Dallas’ death was not noted as a consequence of long-term health challenges that were directly linked to immunosuppressant use. This is ethically problematic because it prevents patient and clinical learning, and inhibits a true picture of FT success measures.

## Data Availability

Data are available upon reasonable request.
